# Nisin Z Potential for the Control of Diabetic Foot Infections Promoted by *Pseudomonas aeruginosa* Persisters

**DOI:** 10.3390/antibiotics12050794

**Published:** 2023-04-22

**Authors:** Rafaela Zina, Eva Cunha, Isa Serrano, Elisabete Silva, Luís Tavares, Manuela Oliveira

**Affiliations:** 1 CIISA—Centre for Interdisciplinary Research in Animal Health, Faculty of Veterinary Medicine, University of Lisbon, Avenida da Universidade Técnica, 1300-477 Lisboa, Portugal; 2 Associate Laboratory for Animal and Veterinary Sciences (AL4AnimalS), 1300-477 Lisboa, Portugal

**Keywords:** diabetic foot infection, nisin Z, persister cells, *Pseudomonas aeruginosa*, transcriptome

## Abstract

Diabetic foot ulcers (DFU) are a major complication of diabetes mellitus and a public health concern worldwide. The ability of *P. aeruginosa* to form biofilms is a key factor responsible for the chronicity of diabetic foot infections (DFIs) and frequently associated with the presence of persister cells. These are a subpopulation of phenotypic variants highly tolerant to antibiotics for which new therapeutic alternatives are urgently needed, such as those based on antimicrobial peptides. This study aimed to evaluate the inhibitory effect of nisin Z on *P. aeruginosa* DFI persisters. To induce the development of a persister state in both planktonic suspensions and biofilms, *P. aeruginosa* DFI isolates were exposed to carbonyl cyanide m-chlorophenylhydrazone (CCCP) and ciprofloxacin, respectively. After RNA extraction from CCCP-induced persisters, transcriptome analysis was performed to evaluate the differential gene expression between the control, persisters, and persister cells exposed to nisin Z. Nisin Z presented a high inhibitory effect against *P. aeruginosa* persister cells but was unable to eradicate them when present in established biofilms. Transcriptome analysis revealed that persistence was associated with downregulation of genes related to metabolic processes, cell wall synthesis, and dysregulation of stress response and biofilm formation. After nisin Z treatment, some of the transcriptomic changes induced by persistence were reversed. In conclusion, nisin Z could be considered as a potential complementary therapy for treating *P. aeruginosa* DFI, but it should be applied as an early treatment or after wound debridement.

## 1. Introduction

Diabetes is a metabolic syndrome characterized by a hyperglycemic state due to decreased insulin secretion, defective insulin activity, or both, and it is considered a chronic global epidemic disease [1]. It is usually associated with foot complications; namely, diabetic foot ulcers (DFUs), which are estimated to affect 25% of all diabetic patients during their lifetime and are very susceptible to the development of infection [2]. Diabetic foot infections (DFIs) are considered one of the most frequent and serious complications of diabetes mellitus [1,2]. DFIs usually progress to involve deep tissues, joints, and bones as a consequence of deranged host defenses mediated by hyperglycemia, neuropathy, and vascular damage, delaying wound healing and leading to an increased likelihood of contamination by opportunistic microorganisms [2]. Thus, proper treatment of infected ulcers is critical, including glycemic control, proper wound care, and administration of antimicrobial agents [3].

The standard treatment approach for DFI includes the physical removal of biofilms via debridement, wound cleansing with an antiseptic solution, and antibiotic therapy [4]. Severe soft tissue infections can be initially treated intravenously with a combination therapy comprising ciprofloxacin and either clindamycin or doxycycline [4]. However, the dissemination of antimicrobial-resistant microorganisms and variants, including persister cells, threatens the effectiveness of antimicrobial therapy, rendering the development of new therapeutic strategies against DFI mandatory [5].

One of the most problematic bacterial species associated with these infections is *Pseudomonas aeruginosa*, a highly versatile bacterium that can easily adapt to complex environments [6]. *P. aeruginosa* is an opportunistic pathogen with a worldwide distribution that belongs to the ESKAPE group of microorganisms and frequently presents a multidrug-resistant profile, and it is associated with high morbidity and mortality rates [7,8]. In fact, *P. aeruginosa* is intrinsically resistant to several antimicrobials, and it is also able to acquire a resistant profile through mutations in chromosomal resistance genes or through the acquisition of resistance determinants from other bacteria [8]. In addition, it is able to express several virulence factors, including adhesins, toxins, exoenzymes, alginate, and biofilms [6,8,9].

Biofilms are complex aggregates of bacteria enclosed in a self-generated matrix of extracellular polymeric substances that protects the bacteria from external aggressions [10,11], and they show high tolerance to antimicrobials and to the action of the host immune system [9,12]. Bacteria present in biofilms exhibit high heterogeneity, as the bacterial subpopulations present in these aggregates may present different rates of metabolic activity [13]. In fact, subpopulations at the biofilm periphery usually exhibit high physiological activity, while subpopulations in the interior exhibit low activity or no growth. The physiological heterogeneity of biofilms affects the activity of many antimicrobials, as a low bacterial metabolic rate may lead to tolerance to antimicrobials due to the inactivity of key targets [7,13]. Accordingly, tolerance seems to be one of the main reasons for the failure of antimicrobials’ action towards biofilms, as a fraction of their cells may survive in the presence of these compounds. This fraction corresponds to a subpopulation of tolerant cells, not mutants, referred to as persister cells, which are phenotypic variants of regular cells that can be present in both *P. aeruginosa* planktonic cultures and biofilms [14,15]. Persisters are non-growing, metabolically quiescent cells that are able to tolerate high concentrations of antimicrobials by reducing their metabolism and entering a dormant state without undergoing genetic changes [11]. As such, persister cells are associated with difficult-to-treat chronic infections [11,16].

Antimicrobial peptides (AMPs) are oligopeptides produced by living organisms as part of their innate immune response against microorganisms, presenting immunomodulatory properties. They have a broad spectrum of antimicrobial action, ranging from viruses to parasites [17,18]. AMPs also exhibit low toxicity and are considered promising candidates for combatting biofilm-associated infections, representing a new therapeutic strategy against DFI [17,18,19]. Nisin is one of the most studied AMPs. Mostly produced by *Lactococcus lactis*, nisin is a cationic bacteriocin with 34 amino acid residues that interacts with the bacterial cell wall precursor lipid II, leading to pore formation [20]. Nisin Z, a nisin variant, has been shown to present inhibitory activity against both Gram-positive bacteria, including *S. aureus* [19,20], most Gram-positive intestinal bacteria [21], *Streptococcus agalactiae*, and coagulase-negative staphylococci [22]; and Gram-negative pathogens, such as *P. aeruginosa* [19]. It shows anti-biofilm activities towards *Listeria monocytogenes* biofilms [23], and it may act synergistically in combination with conventional therapeutic compounds [21], including molecules aiming to destabilize the bacterial outer membrane, such as chelators (e.g., EDTA) [19,22]. When used in combination with another bacteriocin, leucocin C, nisin Z was found to be effective against planktonic *L. monocytogenes* cultures and moderately active against *Salmonella enterica* serovar Enteritidis and *Escherichia coli* [24]. Moreover, a study has shown that multi-component colloidosomes loaded with nisin Z had superior potential for the control of resistant foodborne pathogens, including *Enterococcus faecalis*, in comparison with free nisin Z [25]. Nisin Z also presents antifungal activity, specifically towards *Candida albicans* [26].

Considering the prevalence of *P. aeruginosa* in DFIs and the relevance of persister cells in antimicrobial failure and infection chronicity, this study aimed to evaluate the inhibitory potential of nisin Z towards *P. aeruginosa* persister cells using microtiter plate assays and the differential gene expression in persisters through transcriptomic analysis.

## 2. Results

### 2.1. P. aeruginosa DFI Susceptibility to Ciprofloxacin

The minimum inhibitory concentration (MIC) and the minimum biofilm inhibitory concentration (MBIC) of ciprofloxacin were determined in relation to the *P. aeruginosa* DFI isolate and the reference strain (Table 1).

Ciprofloxacin’s MIC against the DFI isolate was 4.889 ± 0.588 µg/mL, around 20 times higher than that observed for the reference strain (0.250 ± 0 µg/mL). These differences may be related to the fact that the strain under study was isolated from a polymicrobial diabetic foot infection, which provides an optimal environment for resistance transfer and acquisition. Regarding its anti-biofilm activity, ciprofloxacin’s MBIC values for biofilms formed by the *P. aeruginosa* DFI isolate and the reference strain were 2.444 ± 0.294 µg/mL and 0.361 ± 0.044 µg/mL, respectively (Table 1). In a recent study [27], ciprofloxacin’s MIC and MBIC values for both the DFI isolate and the reference strain were higher than ciprofloxacin’s MIC (0.039 µg/mL) and MBIC (0.06 µg/mL) values for *P. aeruginosa* PAO1.

### 2.2. Induction of P. aeruginosa Persister Cells

After chemical treatment with carbonyl cyanide m-chlorophenylhydrazone (CCCP) for 3 h, *P. aeruginosa* suspensions were exposed to the highest ciprofloxacin MIC value (8 µg/mL), allowing an average of 1.525 × 10^5^ cfu/mL persister cells to survive ciprofloxacin treatment.

The highest ciprofloxacin MBIC value (4 µg/mL) did not result in action against non-active cells. The biofilm’s 24 h exposure to this concentration of ciprofloxacin resulted in an average of 1.249 × 10^4^ cfu/mL persister cells.

### 2.3. Persisters’ Susceptibility to Ciprofloxacin

**Figure 1:**
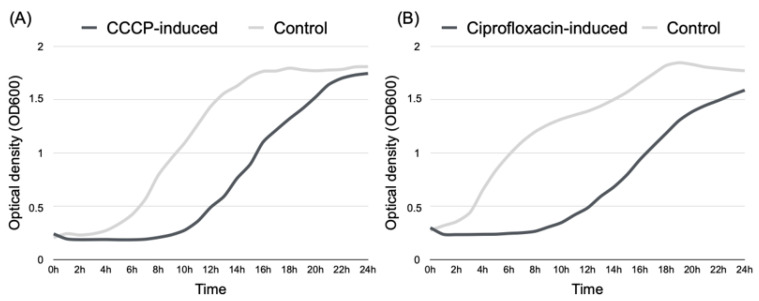
Recuperation of normal growth rate by *P. aeruginosa* Z.25.1 persister cells and growth rate of untreated cultures (control), as determined by optical density (OD 600 nm) in relation to incubation for 24 h. (**A**) CCCP-induced persister cells vs. control; (**B**) ciprofloxacin-induced persister cells vs. control.

To confirm that the persisters obtained were indeed tolerant rather than resistant, their susceptibility to ciprofloxacin was evaluated. After removing the compounds used to induce persistence in *P. aeruginosa* (CCCP and ciprofloxacin), persister cells were exposed to fresh nutrients to allow them to recover their metabolic rate. Cells’ recuperation from a dormant state was confirmed by the optical density, which revealed a long-lasting regrowth lag phase for both CCCP-induced persisters from *P. aeruginosa* planktonic cultures (Figure 1A) and ciprofloxacin-induced persisters from *P. aeruginosa* biofilms (Figure 1B) before cultures eventually resumed a normal growth rate.

After the cells recovered their metabolic rate, they were incubated with different concentrations of ciprofloxacin for determination of MIC and MBIC values in relation to CCCP-induced and CIP-induced persisters, respectively. Results showed that, after recovering, bacterial populations exhibited an antimicrobial-susceptible phenotype, corresponding to MIC and MBIC values of 3.778 µg/mL, lower than those obtained for the control strains used in this assay (*P. aeruginosa* Z25.1 without treatment), for which the MIC value was 5.111 µg/mL and the MBIC value was 4 µg/mL.

### 2.4. Efficacy of Persister Treatment with Nisin Z

The efficacy of nisin Z solutions supplemented with EDTA against *P. aeruginosa* Z25.1 was determined after inducing the formation of persisters in planktonic cultures using CCCP and in biofilms using ciprofloxacin. Results showed that nisin Z + EDTA was able to inhibit CCCP-induced planktonic persisters at nisin concentrations ranging from 5 to 20 μg/mL, with a mean value of 9.167 ± 3.456 μg/mL. However, nisin Z solutions were not able to eradicate persisters present in 24 h biofilms treated with ciprofloxacin, showing an MBIC value of >1250 μg/mL.

### 2.5. Transcriptome Evaluation of Persister Cell Gene Expression

**Figure 2:**
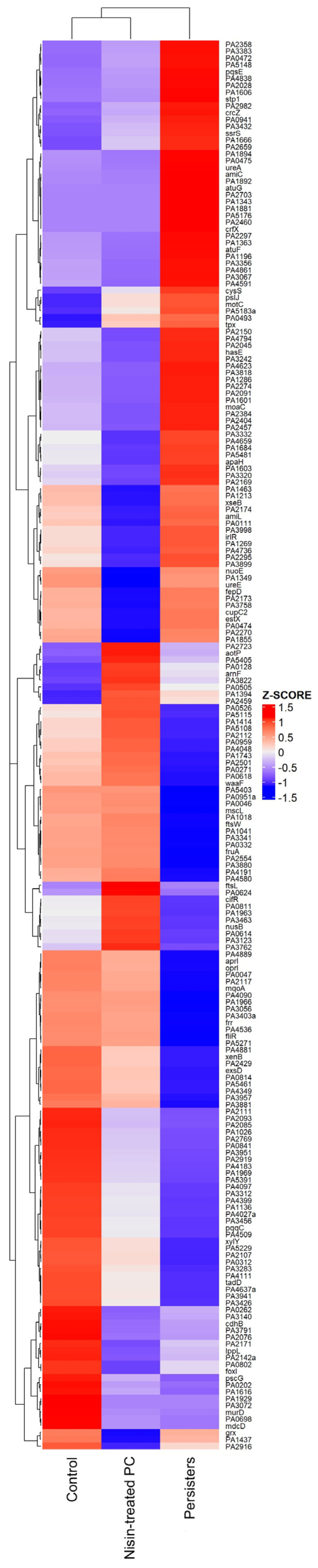
The heat map of the RNA-Seq transcriptome analysis for 207 DEGs from *P. aeruginosa* Z.25.1 isolates, including control cells, persisters, and persister cells (PCs) treated with nisin Z. Colors correspond to per-gene z-scores, ranging from dark blue (1.5), which represents low expression, to dark red (1.5), which represents high expression.

After obtaining *P. aeruginosa* persister cells, the differences at the RNA level between them and the cells of origin were analyzed. RNA-Seq was performed to evaluate the differences between gene expression of control cells (*P. aeruginosa* Z25.1), CCCP-induced persister cells (CCCP + ciprofloxacin), and nisin Z-treated persisters (CCCP + ciprofloxacin + nisin Z). For each pool of three isolates, an average of 25 million high-quality reads were obtained, with an average of 95.4% of sequenced reads successfully aligned to the reference genome of *Pseudomonas aeruginosa* PAO1 ASM676v1. The comparison between control cells, persisters, and nisin Z-treated persisters made it possible to identify a total of 207 differentially expressed genes (DEGs). These are represented in the heat map in Figure 2 and were remarkably distinct, as shown by the different per-gene z-scores.

**Figure 3:**
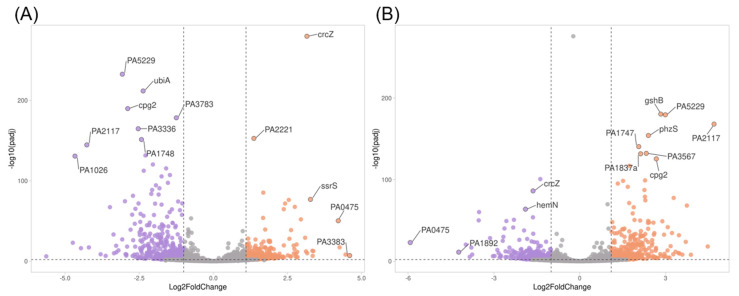
Volcano plot analysis (VolcaNoseR—Exploring Volcano Plots) of DEGs. (**A**) *P. aeruginosa* CCCP-induced persisters vs. control. (**B**) *P. aeruginosa* persisters treated with nisin Z vs. untreated persisters. Downregulated genes are depicted in purple and upregulated genes in orange. Vertical dotted lines indicate a log2 fold-change cut-off above 1 limiting significant differential gene expression. The genes with the highest expression are indicated in the graph.

In comparison with control cells, a total of 655 DEGs (*p*-value < 0.01 and 1 < log2 fold change < 1) were identified in CCCP-induced persisters. From these genes, 371 (57%) were found to be downregulated and 284 (43%) upregulated (Figure 3A). The comparison between gene expression results from untreated persisters and those treated with nisin Z made it possible to identify a total of 587 DEGs, among which 238 (41%) were downregulated and 349 were upregulated (59%) (Figure 3B).

The most downregulated genes (log2 fold change < 2 and *p*-value < 0.01) in persister cells included *ubiA* (4-hydroxybenzoate-octaprenyl transferase) and *cpg2* (carboxypeptidase G2 precursor), while the most upregulated (log2 fold change > 3 and *p*-value < 0.01) were *crcZ* (interferes with Hfq-mediated riboregulation in some strains) and *ssrS* (6S RNA) (Figure 3A).

The most downregulated genes in persisters treated with nisin Z (log2 fold change < 1.5 and *p*-value < 0.01) were *crcZ* and *hemN* (oxygen-independent coproporphyrinogen III oxidase), and the most upregulated (log2 fold change > 2 and *p*-value < 0.01) were *cpg2* (carboxypeptidase G2), *gshB* (glutathione synthetase), and *phzS* (flavin-containing monooxygenase) (Figure 3B).

**Figure 4:**
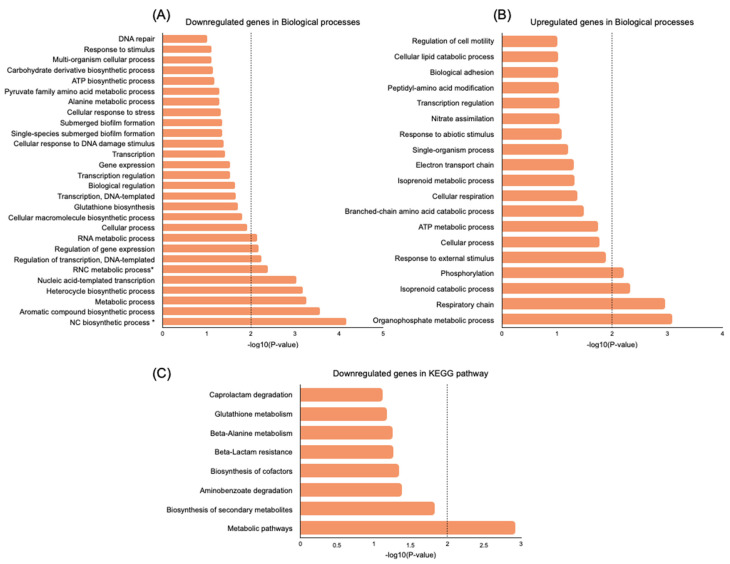
GO and KEGG pathway functional enrichment analysis of significant DEGs in *P. aeruginosa* Z.25.1 persisters vs. control cells. (**A**) Downregulated GO terms. (**B**) Upregulated GO terms. (**C**) Downregulated KEGG pathways. The vertical axis represents the GO or KEGG pathway terms significantly enriched by the DEGs; the horizontal axis indicates the negative log10 value (*p*-value). Legend. * RNC, regulation of nucleobase-containing compound; NC, nucleobase-containing compound.

**Figure 5:**
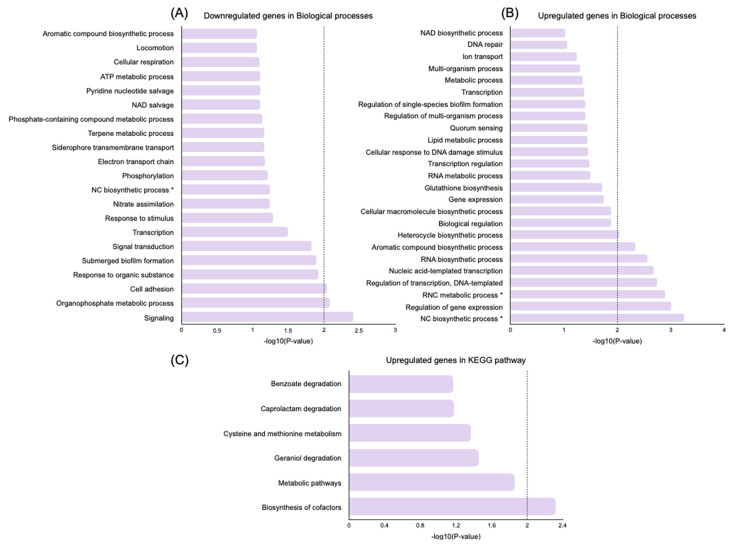
GO and KEGG pathway functional enrichment analysis of significant DEGs in *P. aeruginosa* Z.25.1 persisters treated with nisin Z vs. untreated persisters. (**A**) Downregulated GO terms. (**B**) Upregulated GO terms. (**C**) Upregulated KEGG pathways. The vertical axis represents the GO terms or KEGG pathways significantly enriched by the DEGs; the horizontal axis indicates the negative log10 value (*p*-value). Legend.* RNC, regulation of nucleobase-containing compound; NC, nucleobase-containing compound.

To further explore the biological functions of DEGs, a gene ontology (GO) enrichment analysis and a Kyoto Encyclopedia of Genes and Genomes (KEGG) analysis were performed using the Database for Annotation, Visualization and Integrated Discovery (DAVID) online tool [28]. KEGG pathways and gene-enriched GO terms were identified in the downregulated and upregulated genes induced by persistence (persisters vs. control) and by the treatment of persisters (nisin Z-treated vs. untreated persisters) in the category of biological processes (Figure 4 and Figure 5).

For this analysis, only significant GO terms and KEGG pathways were considered (*p*-value < 0.01).

In persisters vs. control analysis, for *p*-values < 0.01, the downregulated DEGs were enriched in nine biological processes related to biosynthesis of nucleobase-containing compounds and heterocycle and aromatic compounds; metabolic processes, including RNA and nucleobase-containing compound regulation; and gene expression (Figure 4A). The most significant upregulated biological processes (*n* = 4; *p*-value < 0.01) were related to organophosphate metabolic processes, the respiratory chain, the isoprenoid catabolic process, and phosphorylation (Figure 4B). Accordingly, the downregulated DEGs were enriched (*p*-value < 0.01) in metabolic pathways (Figure 4C). Regarding the upregulated DEGs, no statistically significant pathways were found at *p*-values < 0.01.

In nisin Z-treated persisters, the downregulated DEGs were enriched (*p*-value < 0.01) in three significant biological processes related to signaling, the organophosphate metabolic process, and cell adhesion. Moreover, eight biological processes were upregulated (*p*-value < 0.01), which were related to the regulation of nucleobase-containing compounds, gene expression, and transcription, as well as several biosynthetic processes associated with RNA and aromatic and heterocycle compounds (Figure 5A,B). For *p*-values < 0.01, the most significant upregulated pathway was related to biosynthesis of cofactors (Figure 5C), and no significant downregulated pathways were found.

#### Genes Related to Cell Wall Synthesis, Stress Response, Biofilm Formation

Several genes encoding for proteins involved in cell wall synthesis, stress response, and biofilm formation were differentially transcribed between control cells, persisters, and nisin Z-treated persisters (Table 2). The persistence state induced the differential transcription of two genes related to cell wall synthesis (*algk* and *algL*, which were found to be downregulated), three genes related to stress response (*mexR* and *oprN,* downregulated, and *parS,* upregulated), and three genes related to biofilm formation (*lasR,* downregulated, and *pilJ* and *retS* upregulated) (Table 2).

In persisters, the nisin Z treatment induced the differential transcription of four genes related to cell wall synthesis (*algB*, downregulated, and *algL*, *algA*, and *algX,* upregulated), six genes related to stress response (*parS,* downregulated, and *mexR*, *oprF*, *oprG*, *oprI*, and *oprN,* upregulated), and two genes related to biofilm formation (*retS,* downregulated, and *las,* upregulated) (Table 2).

Overall, several of the gene transcription changes induced by persistence were reversed by nisin Z treatment: genes that were found to be downregulated in the persistence state were upregulated in persisters treated with nisin Z (*algL*, *mexR*, *oprN*, and *lasR*), and genes that were found to be upregulated in persistent cells were downregulated after treatment with nisin Z (*parS* and *retS*).

## 3. Discussion

Due to their relation with chronic and relapsing infections, it is important to find alternatives for eliminating the persister cells present in diabetic foot infections [11]. As such, we examined the potential of nisin Z as a new treatment strategy to control persister cells from a *Pseudomonas aeruginosa* clinical strain isolated from a DFI [18].

As Kwan et al. [29] showed, bacterial persistence is induced through environmental pressure and not just through stochastic fluctuations. Different strategies may be used to induce persistence, such as the chemical compound CCCP and the antimicrobial agent ciprofloxacin [11,30]. The generation of persister cells is challenging due to the low number of naturally forming persisters obtained using antibiotic-based methods, as well as the difficulties in separating the surviving persister subpopulation from dead bacteria. Therefore, in this study, CCCP was used to produce persisters only in planktonic cultures. CCCP is a chemical inhibitor of oxidative phosphorylation in mitochondria that uncouples electron transport from ATP synthesis. This compound functions as an ionophore, modifying the transmembrane electrochemical potential and leading to the dispersion of the membrane proton motive force. It reduces the capacity for ATP synthesis, affecting bacterial respiration and respiration-dependent phosphorylation, thus promoting the gradual destruction of living cells and consequent death of the microorganisms [31,32]. Moreover, the reduction in ATP production leads to a drop in metabolic activity, inducing cells to become persisters [11,29]. In this study, both CCCP and ciprofloxacin were able to induce the formation of persister cells, as the exposure of cultures of *P. aeruginosa* in the stationary phase to CCCP allowed the generation of high-efficiency persister cells exhibiting a ciprofloxacin-tolerant phenotype.

Exposure to ciprofloxacin activates bacterial cells’ SOS system, leading to an increase in the formation of persisters; in turn, this SOS response induces several toxin/antitoxin (TA) genes [33,34]. As previously described [34,35], when exposed to ciprofloxacin, bacterial cells induce the expression of toxin TisB, a membrane peptide that causes a decrease in the proton motive force and ATP levels in the membrane, as a response to the ciprofloxacin-induced stress, resulting in dormant and persister cells. Ciprofloxacin-induced persistence in *P. aeruginosa* DFI biofilms yielded 12 times fewer persister colony units than CCCP-induced persistence in planktonic *P. aeruginosa*. The yield difference may be explained by the biofilm’s susceptibility to ciprofloxacin observed in the present study.

Persister cells are considered to be tolerant phenotypic variants rather than resistant cells [35] and are associated with a dormant state. Indeed, our results showed that, after the removal of CCCP and ciprofloxacin, the exposure of induced cells to fresh nutrients allowed them to recover a metabolically active state and an antibiotic-susceptible phenotype, as previously described for persister cells [11].

The development of a persister state is one of the mechanisms associated with increases in antimicrobial resistance [34,35], as well as with the urgent need to develop alternatives to existing treatments, which is especially important for chronic infections such as DFI. Nisin Z has potent antimicrobial efficacy and acts by interacting with the cell membrane and increasing its permeability, causing bacterial death [36,37].

Moreover, it is important to note that, when applied together with the chelator EDTA, nisin Z presents inhibitory activity against DFI isolates, including *P. aeruginosa* [38]. In this study, the antimicrobial potential of nisin Z supplemented with EDTA was evaluated against CCCP-induced persisters from *P. aeruginosa* Z25.1 planktonic cultures and ciprofloxacin-induced persisters from *P. aeruginosa* Z25.1 biofilms. It was observed that nisin Z supplemented with EDTA was able to inhibit persisters in planktonic suspensions but was not able to eradicate biofilms containing the variant phenotype, in spite of its action mechanism being independent of the bacteria’s metabolic state.

To unveil the molecular mechanisms involved in persistence and underlying the effects of nisin Z treatment, a transcriptomic analysis was performed on planktonic *P. aeruginosa* Z25.1 persister cells. Although the transcriptomic changes in *P. aeruginosa* induced by nisin treatment have already been evaluated by different authors [39,40,41,42], none of them have addressed the effect of this AMP on persister cells.

In this study, the gene expression profiles of control cells, persisters, and nisin Z-treated persisters were remarkably different, as can be observed in the heat map (Figure 2), with more than half of the significantly DEGs being downregulated in persisters and more than half of the significantly DEGs being upregulated in nisin Z-treated persisters. Indeed, our results showed that the persistent state (induced by CCCP and ciprofloxacin treatment) promoted more pronounced downregulation of gene expression. The identified downregulated DEGs were enriched in biological processes related to biosynthetic and metabolic processes and gene expression, which was concordant not only with a persistent cell state but also with a decrease in metabolic activity due to the decrease in ATP production caused by CCCP [11,29,34,35]. On the other hand, an increase in the expression of genes associated with respiration and phosphorylation, essential for persisters’ survival, was observed. In accordance, our KEGG pathway analysis revealed that the downregulated genes in persisters were associated with metabolic pathways (*p*-value < 0.01).

These results are not in agreement with those of Murray et al. [42], who found that the eight upregulated genes in PA14 treated with ciprofloxacin (0.5 × MIC) for 30 min were involved in DNA replication, recombination, modification, and repair [42]. This discrepancy may be associated with the different mechanisms of action presented by CCCP and ciprofloxacin, and the treatment period may also have influenced RNA-Seq results.

In nisin Z-treated persisters, when compared to untreated persisters, downregulation of the expression of genes related to quorum sensing and cell adhesion was observed (*p*-value < 0.01), together with upregulation of gene expression and transcription and biosynthetic processes. This upregulation may be involved with changes in the persistence’s metabolic pathways, aiming at counteracting the effect of nisin Z and EDTA. For example, one of the most upregulated genes was *gshB*, which expresses a powerful antioxidant that protects cells from oxidative damage [43,44].

Overall, transcriptomic analysis revealed that several genes involved in *P. aeruginosa*’s response to stress conditions, cell wall synthesis, and biofilm formation were differentially transcribed between the different bacterial groups under study. For example, downregulation of genes associated with cell wall synthesis was detected in the CCCP-induced persisters; namely, *algK* and *algL*, which are involved in the production of alginate by *P. aeruginosa* PAO1, an essential compound for biofilm formation [45,46]. AlgK assists in polymer formation, and AlgL acts as a catalyst of alginate depolymerization as it is also required for alginate production as part of the multi-protein alginate-secretion complex [45]. Therefore, persisters probably produce less alginate and are less adapted to biofilm production and the establishment of chronic infections, which agrees with the findings of Soares et al. [47], who described a great reduction in the production of exopolysaccharides by persister cells.

Although Pagès et al. [48] have shown that CCCP inhibits efflux systems, in this study, MexR, a negative regulator of the MexAB-OpM efflux system, was found to be repressed in CCCP-induced persisters [49]. Likewise, ParS, involved in the ParS/ParR system, which regulates *P. aeruginosa* efflux genes and porin expression, was found to be overexpressed [50]. These results support the hypothesis that this bacterial species can express several efflux systems with overlapping properties [48].

Biofilm structures act as a diffusion barrier that limits access to antimicrobials, and their regulation depends mostly on quorum-sensing (QS) systems, two-component regulatory systems, exopolysaccharides, and c-di-GMP [51]. The QS system LasI-LasR contributes to the formation of mature and differentiated biofilms, and the sensory kinase RetS of the two-component system GacA/GacS senses environmental stimuli, contributing to the lifestyle adaptations of bacterial populations [51,52]. In this study, the downregulation of *lasR* and upregulation of *retS* observed in CCCP-induced persisters suggested that, in a persistence state, cells do not promote biofilm formation and are less adapted to the development of chronic infections [52].

In the persister cells treated with nisin Z, the action of this antimicrobial seems to have influenced membrane permeability and the expression of efflux pump- and biofilm-related genes. In these cells, the vast majority of cell wall synthesis genes were found to be upregulated, suggesting that they can produce more alginate than untreated cells, which may lead to lower membrane permeability [45]. Furthermore, the upregulation of several genes encoding outer membrane proteins (*oprF*, *oprG*, *oprI*) suggests that nisin Z treatment induces a decrease in membrane permeability [53]. This decrease, associated with the observed upregulation of the multidrug efflux protein gene *oprN*, as well as *phzS* and *gshB*, suggests that the reaction of cells regarding nisin treatment was towards hampering nisin Z entrance to the cells and decreasing its effects, which is related to defense against enhanced oxidative injury [43,44]. Nisin Z treatment also induced changes in the expression of biofilm-related genes by *P. aeruginosa* Z25.1, as *lasR* was found to be upregulated and *retS* downregulated. This result suggests that the ability of persister cells to promote biofilm formation and chronic infection increases after exposure to nisin Z [52]. However, the *mexR* overexpression and *parS* downregulation detected in nisin Z-treated persisters suggest that these cells are susceptible to nisin Z, which indicates that this AMP can still be considered for DFI treatment.

One of the limitations of this study was that transcriptomic evaluation was only performed using planktonic persisters, and what happens in the gene expression profiles of persisters in established biofilms may not be in accordance with the results observed. In any case, this study provides insights into the number and types of pathways possibly altered in persister populations before and after treatment with nisin Z. Furthermore, making inferences from these data to what happens in vivo should be undertaken with caution, as several pathways, including numerous efflux systems, may have higher expression in in vivo infections compared to in vitro cultures, whereas other metabolic pathways, such as the tricarboxylic acid (TCA) cycle, can be downregulated in human samples [54].

## 4. Materials and Methods

### 4.1. Bacterial Strains

The biofilm-producing strain *P. aeruginosa* Z25.1, previously isolated from a DFI and characterized by our team, was used in this study [55]. The *P. aeruginosa* ATCC 27583 reference strain was included as a control. Bacterial isolates were stored at −80 °C until use.

### 4.2. Stock Solutions

Ciprofloxacin stock solution (2 mg/mL) was obtained by dissolving ciprofloxacin (Sigma-Aldrich, St. Louis, MO, USA) in 0.1 N HCl (Merck, Darmstadt, Germany). The solution was filtered (0.22 µm filter, Millipore, Burlington, MA, USA) and stored at −80 °C. Working solutions of ciprofloxacin were prepared in sterile Milli-Q water at concentrations ranging from 0.125 to 256 µg/mL and stored at 4 °C.

Nisin Z stock solution was prepared at 5 mg/mL by dissolving ultrapure nisin Z (Handary, Brussels, Belgium) in sterile Milli-Q water. The solution was filtered (0.22 µm filter) and stored at 4 °C. Working solutions of nisin Z were diluted in sterile Milli-Q water at concentrations ranging from 2.5 to 1250 µg/mL and stored at 4 °C.

In the persister susceptibility assays, nisin solutions were supplemented with EDTA at 4000 µg/mL, as previously described by us [38]. Thus, an EDTA stock solution was prepared at 16 mg/mL by dissolving EDTA (Sigma-Aldrich, Darmstadt, Germany) in sterile Milli-Q water, and stored at 4 °C.

The carbonyl cyanide m-chlorophenylhydrazone (CCCP) stock solution was prepared at 40 mg/mL according to the manufacturer instructions by dissolving CCCP (Sigma-Aldrich, St. Louis, MO, USA) in dimethyl sulfoxide (DMSO) (Sigma-Aldrich, St. Louis, MO, USA) and stored at −20 °C. Working solutions of CCCP were diluted in DMSO at a concentration of 200 µg/mL and stored at −20 °C.

### 4.3. Determination of the Minimum Inhibitory Concentration (MIC) and Minimum Biofilm Inhibitory Concentration (MBIC) of Ciprofloxacin towards P. aeruginosa

To determine ciprofloxacin’s MIC for the isolates under study [56], which was necessary to select the ciprofloxacin concentration to be applied later in this study (Section 2.3 and Section 2.4), 50 µL serial dilutions of ciprofloxacin ranging from 0.125 to 256 µg/mL were distributed in the wells of 96-well microtiter plates. Each well was then filled with 150 µL of *P. aeruginosa* bacterial suspensions at 10^5^ cfu/mL prepared by dilution in fresh Mueller-Hinton broth (MHb, VWR Chemicals, Leuven, Belgium). Control wells were filled with 200 µL of fresh MHb (negative control) or 200 µL of *P. aeruginosa* suspension (positive control). The plates were incubated at 37 °C for 24 h, after which MIC values were determined as the lowest concentration of ciprofloxacin that visually inhibited bacterial growth. Each isolate was evaluated in triplicate wells with three independent assays.

The inhibitory activity of ciprofloxacin against *P. aeruginosa* biofilms was evaluated by determining the MBIC [56]. Briefly, bacterial suspensions were diluted in fresh tryptic soy broth (TSB) (VWR Chemicals, Leuven, Belgium) supplemented with 0.25% glucose to a final concentration of 10^5^ cfu/mL. Then, 200 µL portions of these bacterial suspensions were transferred to the wells of 96-well microtiter plates, which were covered with 96-peg lids and incubated at 37 °C for 24 h to allow biofilm formation on the pegs. Peg-lids were sequentially washed in 0.9% sodium chloride (NaCl) and placed on new microtiter plates containing fresh medium either supplemented with different concentrations of ciprofloxacin ranging from 0.125 to 256 µg/mL or without ciprofloxacin and incubated at 37 °C for 24 h. After incubation, MBIC values were determined as the lowest ciprofloxacin concentration that visually inhibited bacterial growth in the suspensions of the microtiter plates. Each isolate was evaluated in triplicate wells with three independent assays.

### 4.4. Induction of Persister Cells in P. aeruginosa Planktonic Cultures using CCCP

Each isolate was inoculated in Luria-Bertani medium (LB) (VWR Chemicals, Leuven, Belgium) and incubated with shaking (180 rpm) for 18 h at 37 °C to obtain stationary phase cultures. Thereafter, cultures were incubated with CCCP at 200 µg/mL for 3 h at 37 °C, with shaking [11].

The ability of CCCP to induce a persistence phenotype was confirmed by assessing survival after antibiotic treatment [11]. Briefly, after treatment with CCCP, bacteria were washed twice with phosphate-buffered saline (10× PBS, pH 7.4; 1700× *g* for 10 min) and re-suspended in PBS/LB (PBS supplemented with 1% of LB) at a final density of ≈10^6^ cfu/mL. Bacterial suspensions were then exposed to the highest MIC of ciprofloxacin (8 µg/mL) for 3 h. After antibiotic treatment, bacteria were inoculated on tryptic soy agar (TSA) medium (VWR Chemicals, Leuven, Belgium), and incubated for 48 h at 37 °C, following which the number of colony-forming units among the surviving persister cells was determined. Untreated bacteria and bacteria treated with CCCP but without incubation in the presence of the antibiotic were used as controls. Three independent assays were performed in duplicate.

Moreover, the ability of CCCP to induce persistence was also confirmed by determining ciprofloxacin’s MIC. Bacterial suspensions were incubated as described and bacterial growth monitored via OD600 measurements (FLUOstar Omega OPTIMA, BMG LABTECH, Orten-berg, Germany) every hour for 24 h. Bacteria were harvested at the early exponential phase growth and 150 µL portions of the 10^6^ cfu/mL suspensions were placed in the wells of 96-well microtiter plates either containing 50 µL ciprofloxacin suspensions with different concentrations (0.125–256 µg/mL) or in the absence of the antimicrobial agent [11]. MIC values were defined as the lowest concentration of ciprofloxacin that resulted in complete inhibition of visible growth after 24 h of incubation at 37 °C.

### 4.5. Induction of Persister Cells in P. aeruginosa Biofilms Using Ciprofloxacin

For biofilm formation, isolates were first inoculated in TSB and incubated for 18 h at 37 °C. After incubation, bacterial suspensions were diluted (1:100) in TSB supplemented with 0.25% glucose, following which 200 µL was distributed in the wells of a 96-well microtiter plate covered with a 96-peg lid and incubated for 24 h at 37 °C [30]. To induce the formation of persisters, a 24 h old biofilm was treated with a previously defined concentration of ciprofloxacin (4 µg/mL) for 24 h and incubated at 37 °C [30]. Biofilms without ciprofloxacin treatment were used as a control. Three independent assays were performed using triplicate wells.

The ability of ciprofloxacin to induce a persistence phenotype within *P. aeruginosa* biofilms was evaluated by determining ciprofloxacin’s MBIC. Thus, 24 h old biofilms were treated with 4 µg/mL of ciprofloxacin for 24 h at 37 °C, washed in NaCl 0.9%, and placed in new microtiter plates with fresh medium. Bacterial growth was monitored via OD600 measurements (FLUOstar Omega OPTIMA) every hour for 24 h. Bacteria were harvested at the early exponential growth phase, and 10^6^ cfu/mL suspensions were distributed as described in 96-well microtiter plates with either different concentrations of ciprofloxacin ranging from 0.125 to 256 µg/mL or without ciprofloxacin and incubated for 24 h at 37 °C. MBIC values were defined as the lowest concentration of ciprofloxacin at which there was no time-dependent increase in the mean number of biofilm-viable cells. Three independent assays were performed.

### 4.6. Evaluation of the Nisin Z Activity against Planktonic and Biofilm Persister Cells

Both the MIC and the minimum biofilm eradication concentration (MBEC) of nisin Z were evaluated in relation to planktonic and biofilm persisters, respectively.

The antimicrobial activity of nisin Z was tested against planktonic persister cells induced with CCCP. Treated and untreated bacteria were diluted in PBS/LB to a final concentration of ≈10^6^ cfu/mL and 150 µL portions of these suspensions were incubated with 50 µL of nisin Z solutions at different concentrations (2.5 to 1250 µg/mL) supplemented with EDTA (4000 µg/mL) using a 96-well microtiter plate. Bacterial suspensions incubated in the absence of AMP+EDTA were used as a control. After 3 h of incubation at 37 °C, the suspensions were diluted and plated on TSA for determination of colony-forming units.

The antimicrobial activity of nisin Z was also evaluated against biofilm persister cells induced with ciprofloxacin. Briefly, cells from a 24 h old biofilm established in a peg-lid, treated with ciprofloxacin for 24 h and without treatment, were washed three times in NaCl 0.9%. Then, the peg-lid was transferred to a new microtiter plate filled with nisin Z (2.5 to 1250 µg/mL) supplemented with EDTA (4000 µg/mL) and incubated for 24 h at 37 °C. Biofilms incubated in the absence of AMP+EDTA were used as control. After incubation, cells were washed three times in NaCl 0.9% and placed in a new microtiter plate with TSB + 0.25% glucose and sonicated for 15 min at 50 Hz. Then, the peg-lid was discarded and replaced with a normal lid, and the microtiter plate was incubated for 24 h at 37 °C. After incubation, MBEC values, defined as the minimum concentration of nisin Z able to inhibit bacterial growth after exposing persisters to fresh nutrients, were determined. Three independent assays were performed.

### 4.7. RNA Extraction, Sequencing, and Data Analysis

RNA from three replicates of the bacterial suspensions of *P. aeruginosa* Z25.1 planktonic persister cells treated with nisin Z (CCCP+ ciprofloxacin + nisin Z 2.5 µg/mL), of *P. aeruginosa* Z25.1 planktonic persister cells not treated with nisin Z (CCCP+ ciprofloxacin), and of *P. aeruginosa* Z25.1 planktonic cells was extracted using the miRNeasy kit according to the manufacturer’s instructions.

RNA sequencing was performed by BioISI—Biosystems and Integrative Sciences Institute. Briefly, library preparation was performed with prokaryotic rRNA depletion using the Illumina stranded library kit and paired-end sequencing was performed on an Illumina NextSeq 500. Differential expression analysis was performed using sequencing reads alignment against *Pseudomonas aeruginosa* PAO1 reference genome ASM676v1, followed by employment of sequence alignment and map (SAM) files containing information for each individual read and its alignment to the genome. SAM files were randomly split into three independent files before the read count to allow differential expression analysis, which was performed with gene count tables using R Bioconductor package DESeq2. Additionally, only statistically significant genes were analyzed—i.e., those with a baseMean greater than 10—in order to exclude noise, and the genes also had *p*-values < 0.01 and a log2 fold change cut-off above 1. Volcano plots and heat maps of the RNA-Seq transcriptome analysis, as available in the R Bioconductor package DESeq2, were used for quality control and to generate plots of expression.

In order to analyze the screened differentially expressed genes (DEGs) at the functional level, gene ontology (GO) enrichment and Kyoto Encyclopedia of Genes and Genomes (KEGG) pathway analysis were performed using the DAVID Database for Annotation, Visualization and Integrated Discovery (DAVID 6.8, https://david.ncifcrf.gov/, accessed on 30 January 2023) online tool. DAVID is an online program that may be used to systematically extract biological meaning from large lists of genes or proteins [28]. The standard parameters of DAVID were used as the cut-off criteria.

## 5. Conclusions

In this study, nisin Z presented a high in vitro inhibitory efficacy against *P. aeruginosa* DFI planktonic persisters at low concentrations (5–20 μg/mL). However, it was unable to eradicate persister cells in established biofilms at the concentrations tested, as the cells could adapt to stressful conditions by decreasing outer membrane permeability or increasing the expression of efflux pumps, impairing antimicrobial action.

Overall, the transcriptomic analysis identified potential persistence-associated biomarkers and highlighted the molecular mechanisms underlying the effects of nisin Z treatment. As expected, the persistence state was associated with downregulation of genes related to metabolic processes, which was in accordance with the cells’ state of dormancy. The transcriptome of persisters also revealed downregulation of genes involved in cell wall synthesis and dysregulation of genes related to stress response and biofilm formation.

As the transcription of genes involved in membrane permeability was downregulated, it can be suggested that, after CCCP treatment, cells may have gained higher membrane permeability, which may have facilitated the entrance of both CCCP and ciprofloxacin, enabling the production of persisters. Furthermore, results showed that CCCP-induced persisters may be less able to form biofilms and less adapted to the establishment of chronic infections. After treatment with nisin Z, persisters demonstrated reversal of some of the transcriptomic changes induced by persistence, mainly in genes related to cell wall synthesis, stress response, and biofilm formation. In fact, after treatment, the transcription profile suggested that persisters lost membrane permeability, which may have hampered nisin Z entrance. Further, the transcription profile of biofilm-related genes revealed that nisin Z treatment induced biofilm formation by persisters, which may be associated with chronic infections. Overall, these results suggest that nisin Z supplemented with EDTA can complement conventional therapies used to treat severe *P. aeruginosa* diabetic foot infections and that it should be applied as an early treatment or after wound debridement.

## Figures and Tables

**Table 1:** MIC and MBIC of ciprofloxacin against *P. aeruginosa* Z25.1 and *P. aeruginosa* ATCC 27583. Data shown as means ± SD; each group value is an average of three independent measurements.

	*P. aeruginosa* Z25.1	*P. aeruginosa* ATCC 27583
MIC	4.889 ± 0.588 µg/mL	0.250 ± 0 µg/mL
MBIC	2.444 ± 0.294 µg/mL	0.361 ± 0.044 µg/mL

**Table 2:** DEGs in persister cells vs. control cells and in *P. aeruginosa* Z25.1 persisters treated with nisin Z vs. untreated cells. All genes that showed significant differential expression are listed with adjusted *p*-values < 0.01 and log2 fold changes of >1.

Condition	Biological Process	Gene	Functional Protein	Log_2_ Fold Change (*)
PCs vs. C	Cell wall synthesis	*alg*K	Alginate biosynthesis protein	−1.96
		*alg*L	Alginate lyase	−2.23
	Stress response	*mex*R	Multidrug resistance operon repressor	−1.12
		*opr*N	Multidrug efflux outer membrane protein	−1.27
		*par*S	Two-component sensor	1.59
	Biofilm formation	*las*R	Transcriptional regulator	−1.7
		*pil*J	Twitching motility protein	1.18
		*ret*S	Sensor histidine kinase MifS	1.41
NPCs vs. PCs	Cell wall synthesis	*alg*B	Alginate biosynthesis transcriptional regulatory protein AlgB	−1.15
		*alg*L	Alginate lyase	1.97
		*alg*P	Transcriptional regulatory protein ALgP	1.08
		*alg*X	Alginate biosynthesis protein AlgX	1.01
	Stress response	*mex*R	Multidrug resistance operon repressor	1.49
		*opr*F	Outer membrane porin F	1.7
		*opr*G	Outer membrane protein OprG	1.14
		*opr*I	Outer membrane lipoprotein I	3.57
		*opr*N	Multidrug efflux outer membrane protein	1.75
		*par*S	Two-component sensor	−1.68
	Biofilm formation	*las*R	Transcriptional regulator	1.45
		*ret*S	Sensor histidine kinase MifS	−2.04

Legend: (*) Positive log2 fold-change values indicate genes with higher transcript levels; negative values indicate genes with lower transcript levels. PC, persister cell; NPC, nisin Z-treated persister cell; C, control.

## Data Availability

The data presented in this study are available on request from the corresponding author. The data are not publicly available due to privacy reasons.

## Abbreviations

diabetic foot ulcer	DFU
diabetic foot infection	DFI
carbonyl cyanide m-chlorophenylhydrazone	CCCP
antimicrobial peptides	AMPs
minimum inhibitory concentration	MIC
minimum biofilm inhibitory concentration	MBIC
minimum biofilm eradication concentration	MBEC
differentially expressed genes	DEGs
gene ontology	GO
Kyoto Encyclopedia of Genes and Genomes	KEGG
Database for Annotation, Visualization and Integrated discovery	DAVID
dimethyl sulfoxide	DMSO
Mueller-Hinton broth	MHb
tryptic soy broth	TSB
sodium chloride	NaCl
Luria-Bertani medium	LB
phosphate-buffered saline	PBS
tryptic soy agar	TSA
sequence alignment and map	SAM
